# On Hepatitis C Virus Evolution: The Interaction between Virus and Host towards Treatment Outcome

**DOI:** 10.1371/journal.pone.0062393

**Published:** 2013-04-25

**Authors:** Cíntia Bittar, Ana Carolina Gomes Jardim, Lilian Hiromi Tomonari Yamasaki, Claudia Márcia Aparecida Carareto, João Renato Rebello Pinho, Philippe Lemey, Isabel Maria Vicente Guedes de Carvalho-Mello, Paula Rahal

**Affiliations:** 1 Department of Biology, UNESP – São Paulo State University – IBILCE­ – Institute of Bioscience, Language & Literature and Exact Science, São José do Rio Preto, São Paulo, Brazil; 2 Department of Gastroenterology – Laboratory of Hepatology and Gastroenterology from Institute of Tropical Medicine, USP – São Paulo University – Faculty of Medicine, São Paulo, São Paulo, Brazil; 3 Department of Clinical Pathology, Albert Einstein Israeli Hospital, São Paulo, São Paulo, Brazil; 4 Katholieke Universiteit Leuven - Laboratory of Clinical and Epidemiological Virology (Rega Institute), Leuven, Belgium; 5 Departamento de Medicina - Disciplina de Gastroenterologia, Laboratório de Hepatologia Molecular Aplicada, Universidade Federal de São Paulo - Escola Paulista de Medicina, São Paulo, São Paulo, Brazil; National Institute of Allergy and Infectious Diseases, United States of America

## Abstract

**Background:**

Hepatitis C is a disease spread throughout the world. Hepatitis C virus (HCV), the etiological agent of this disease, is a single-stranded positive RNA virus. Its genome encodes a single precursor protein that yields ten proteins after processing. NS5A, one of the non-structural viral proteins, is most associated with interferon-based therapy response, the approved treatment for hepatitis C in Brazil. HCV has a high mutation rate and therefore high variability, which may be important for evading the immune system and response to therapy. The aim of this study was to analyze the evolution of NS5A quasispecies before, during, and after treatment in patients infected with HCV genotype 3a who presented different therapy responses.

**Methods:**

Viral RNA was extracted, cDNA was synthesized, the NS5A region was amplified and cloned, and 15 clones from each time-point were sequenced. The sequences were analyzed for evolutionary history, genetic diversity and selection.

**Results:**

This analysis shows that the viral population that persists after treatment for most non-responder patients is present in before-treatment samples, suggesting it is adapted to evade treatment. In contrast, the population found in before treatment samples from most end-of-treatment responder patients either are selected out or appears in low frequency after relapse, therefore changing the population structure. The exceptions illustrate the uniqueness of the evolutionary process, and therefore the treatment resistance process, in each patient.

**Conclusion:**

Although evolutionary behavior throughout treatment showed that each patient presented different population dynamics unrelated to therapy outcome, it seems that the viral population from non-responders that resists the treatment already had strains that could evade therapy before it started.

## Introduction

Hepatitis C is a world-wide disease. The World Health Organization estimate is that 3% of the world’s population has been infected with the hepatitis C virus (HCV), which is the etiological agent of this disease [1].

HCV is a single-stranded positive RNA virus member of the Flaviviridae family. Its genome is 9.6 Kb long and encodes for a single precursor protein with approximately 3,000 amino acid residues. This polypeptide is processed by viral and host proteins, resulting in 10 individual proteins: core, E1, E2, p7, NS2, NS3, NS4A, NS4B, NS5A and NS5B [2].

NS5A, one of the viral non-structural proteins, is the protein most associated with interferon-based therapy, the accepted treatment for hepatitis C. NS5A is multifunctional, and although not all of its functions are clearly established yet, it is known to be involved in viral replication and interactions with cell signaling pathways [3], [4], [5].

Some studies have identified regions in NS5A that have specific functions. At the amino terminal end there is a 27 amino acid cytoplasmic retention signal (CRS) responsible for keeping NS5A in the cytoplasm [6]. Another region, called the PKR-binding region, is responsible for binding to cellular protein kinase R (PKR), inhibiting it, and ultimately resulting in the suppression of interferon (IFN) antiviral activity [7]. Within the PKR-binding region there is an IFN sensitivity-determining region (ISDR), which some studies correlated the accumulation of mutations to therapy response [8], [9]. Despite the CRS, NS5A also contains a nuclear localization signal (NLS) that, in the absence of CRS, results in the translocation of NS5A to the nucleus [6], [10]. In the C-terminal portion of NS5A the genetic variability of a region called V3 is also associated with IFN therapy response [11], [12].

Owing to the lack of proof-reading activity of the viral RNA-dependent RNA polymerase (NS5B protein), HCV has a high mutation rate and therefore high variability. This variation happens at different levels, including genotypes (30% to 35% difference), subtypes (20% to 25% difference) and different but closely related genomes called quasispecies [13], [14], [15]. Although mutations can be prejudicial for the virus when they result in non-viable strains, having a pool of slightly different strains with mutations that are initially neutral or quasi-neutral can increase fitness after changes in the initial condition, and be useful for evading the immune response and treatment [16], [17], [18], [19].

The aim of this study was to analyze the evolution of NS5A quasispecies before, during, and after treatment of patients infected with HCV genotype 3a, which presents different therapy responses. This is the first study to analyze the evolutionary history, genetic diversity and selection of full-length NS5A quasispecies from HCV genotype 3a throughout treatment.

## Results

### Sequences

This study generated 435 sequences of full length NS5A (1,356 bp) from samples collected during and after interferon plus ribavirin treatment from 8 patients,4 Non-responder (NR) and 4 End-of-treatment responder (ETR). The sequences were submitted to GenBank; their accession numbers are **JN689511–JN689930**. Sequences from samples collected before treatment from these 8 patients and also from 4 Sustained virological response patients that were previously published [20] were used in analysis in order to analyze the evolutionary behavior throughout time-points (see Materials and Methods section for more details). The total population of study used on the analysis is 12 patients.

### Quasispecies Analysis

**Figure 1:**
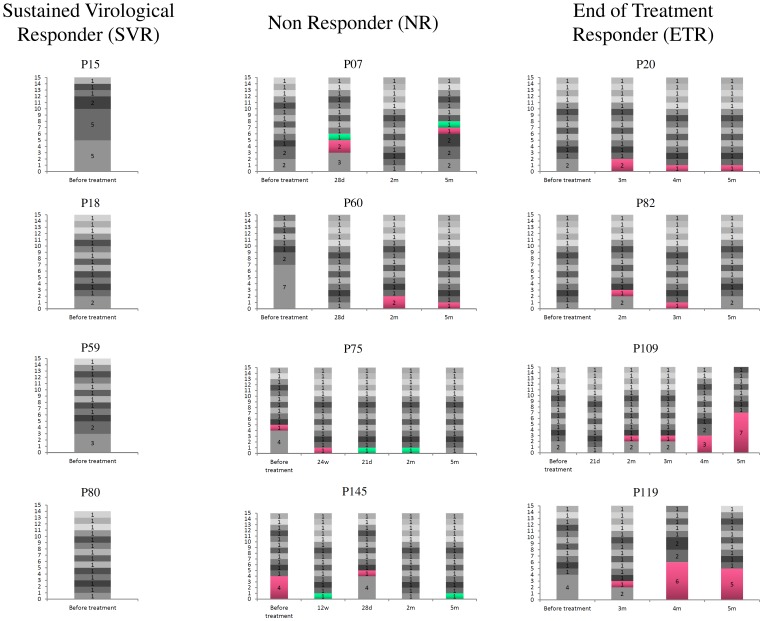
Eschematic representation of quasispecies. Left Y axis - graphic representation of different amino acid variants. Colored tiles represent the same variant. w-weeks; d-days; m-months. SVR data previously published in Bittar et al 2010 [20].

Sequences were analyzed to address quasispecies variability at each time point in each patient. Figure 1 shows a graphic representation of the results; each tile represents a different amino acid sequence and colored tiles represent a persistent sequence through time points. The viral quasispecies analysis from patient P75 at 24 weeks (w) of treatment and in patient P145 at 28 days (d) after treatment, both non-responder patients, shows that amino acid sequences from strains that were circulating before treatment were also found during and after treatment (Figure 1). Patients P07, P60 and P75 had sequences that were sampled at more than one time-point after treatment. Patient P07 presented two strains that were sampled at the 28 days (d) and 5 month (m) time-points, patient P60 presented one sequence that was found at both 2 months and 5 months, and patient P75 had the same sequence at the 21 day and 2 month time-points. Patient P145 presented one sequence that was first sampled at 12 weeks (w) during the treatment, then later sampled at 5 months.

In contrast, most end-of-treatment responder patients had a different profile. No variants from before treatment were found during or after treatment. However, it was shown that a strain that arises at the relapse time-point, or close to the relapse point, such as in patient P109 (2 m), is sampled until the end of the follow-up, with the exception of patient P82. In two patients, P109 and P119, this sequence increased in frequency with increasing time (Figure 1).

### Genetic Distance

**Figure 2:**
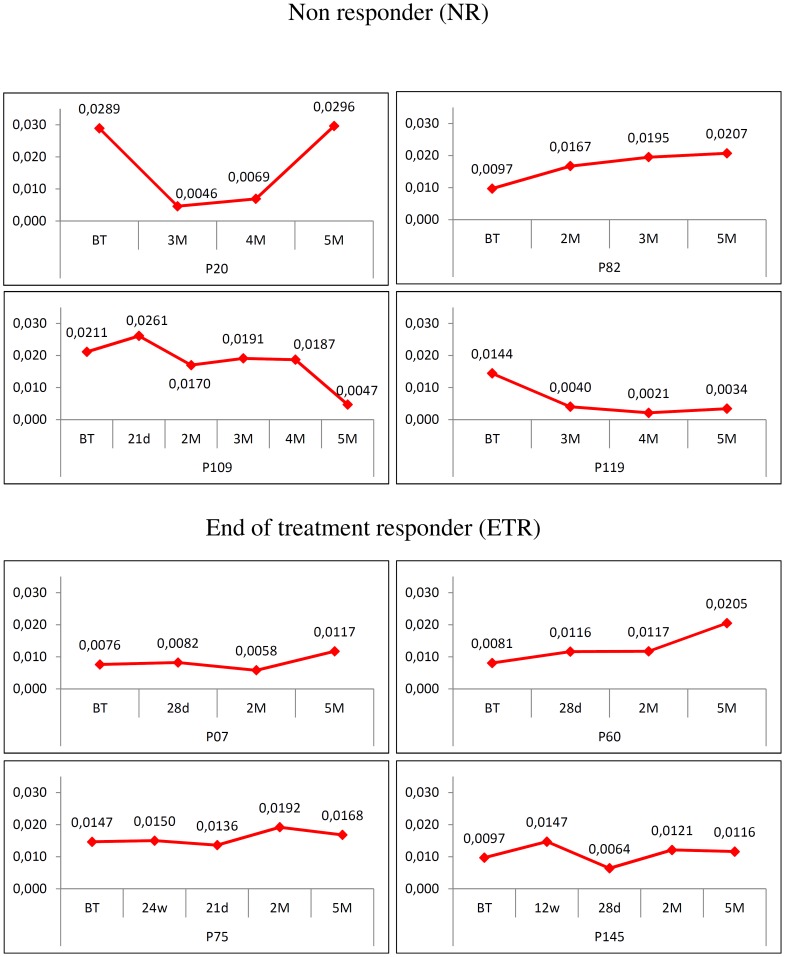
Genetic distance. Genetic distances calculated by the number of base differences per site from between sequences within each time point for each patient. BT-Before treatment; w-weeks; d-days; M-months.

Genetic distance data, calculated within each time-point, shows that the non-responder patients presented a more homogeneous quasispecies during treatment than the end-of-treatment responder patients, except for patient P60 whose the genetic distance was 0.0081 before treatment, and then rose through follow-up to 0.0205 at 5 months (Figure 2). Quasispecies in the end-of-treatment responder patients showed variable genetic distances and different behaviors depending upon the patient. Patient P20, for example, although presenting quasispecies with similar distances in before-treatment (0.0289) and 5 month time-points (0.0296) – difference was not statistically significant, showed much lower distances at 3 and 4 months (0.0046 and 0.0069, respectively). In patient P82, the genetic distance rose significantly from 0.0097 to 0.0207, stabilizing at 3 and 5 months (no significance); in contrast, in patient P119 it decreased from 0.0144 to 0.0034 (statistically significant). Patient P109 also showed a significant decrease in genetic distance from the before-treatment (0.0211) to 5 month (0.0047) time-points. However, at 21 days after treatment (relapse point) the distance was higher (0.0261) than before treatment; at 2 months it started falling (0.0170) and remained constant until 4 months, then it showed a drastic decrease at the 5 month time-point (0.0047) (For detailed statistics see Table S1).

### Global and site-by-site Selective Pressure (ω) Estimation

**Figure 3:**
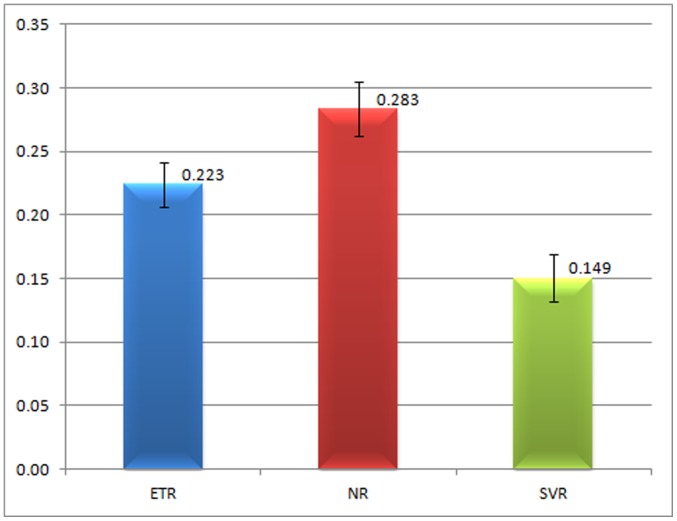
Overall ω. Overall ω of each response group. ETR: end-of-treatment responder; NR: non-responder; SVR: sustained virological responder.

The estimates of the overall selective pressure (ω = dN/dS) of each of the responder groups showed that non-responder patients have a similar ω (0.2828) to the end-of-treatment responders (0.2234). This analysis was also carried out for the sequences from the sustained virological responder group (**EU826189 to EU826218; EU826249 to EU826263; EU826294 to EU826307**), which presented lower ω values (0.1494) (Figure 3), although the differences were not statistically significant.

**Figure 4:**
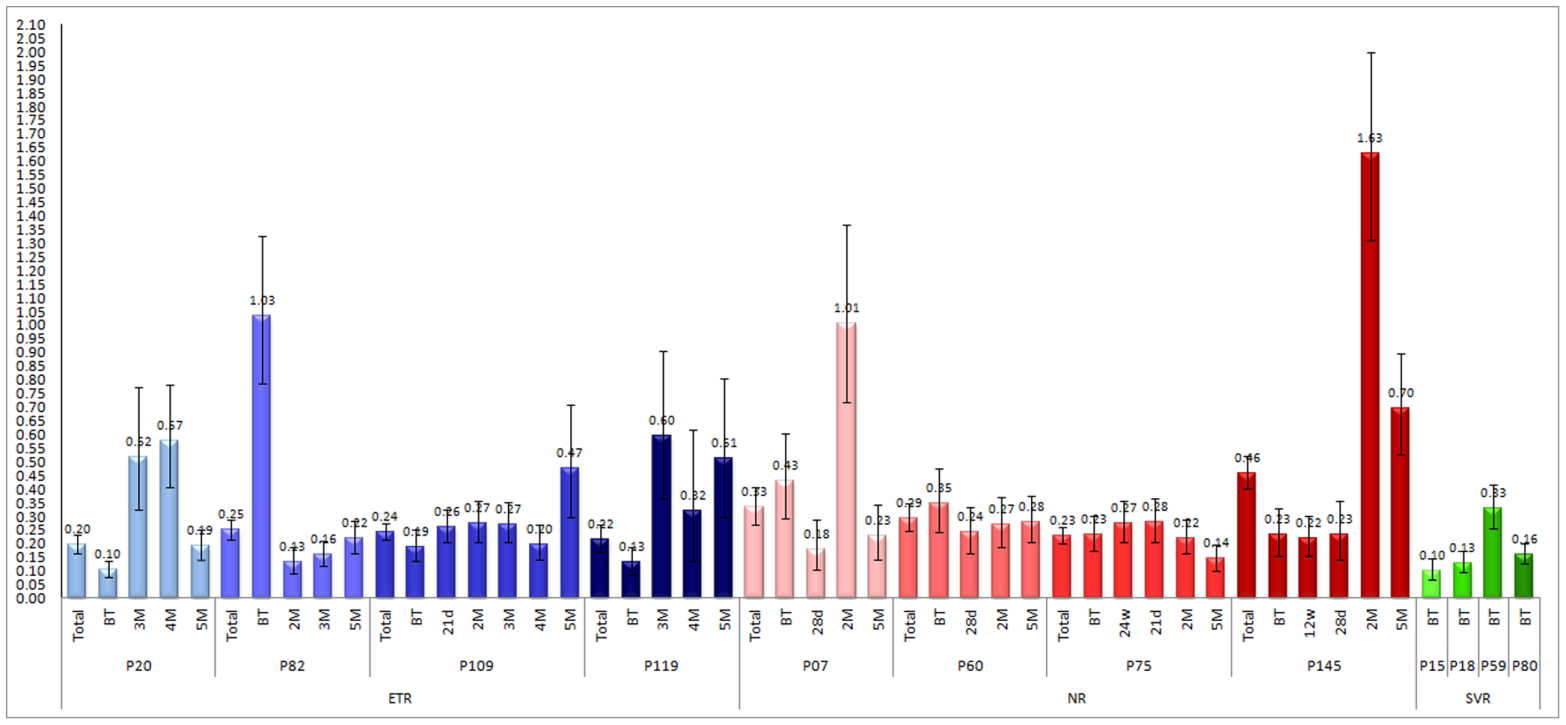
ω per patient. ω rates for each patient (Total) and for each time point. BT-Before treatment; w-weeks; d-days; m-months.

When ω was analyzed by patient, the values varied from 0.20 to 0.46, suggesting that NS5A is under a relaxed purifying selection in the quasispecies circulating in the patients. However, by analyzing each time-point separately, variations could be detected. Patient P145 was the only individual who showed evidence of positive selection at 2 months, with ω = 1.63, but by 5 months this had decreased to 0.7. Evidence of neutral evolution was found in time-points from two patients, P82– BT (Before treatment) and P07–2 m, with ω values of 1.03 and 1.01 respectively (Figure 4).

**Figure 5:**
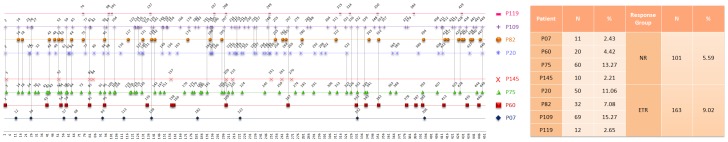
Negative selection. Sites under negative selection for each patient. Measured by ω rates considering p<0.1. Vertical lines link sites negatively selected in more than one patient.

The estimation of the dN/dS rates for each site showed negatively selected sites in the NS5A sequences of all patients, in agreement with the above results. Figure 5 indicates the locations of these sites in the sequence and their percentages in each patient. However, evidence for positive selection was found in two amino acids, 122 and 408 of the strains from patient P109. Negatively selected sites were identified in all patients. The results are shown in Figure 5. End-of-treatment responder patients had more negatively selected sites (n−163; 9.02%) than non-response patients (n−101; 5.59%), and the difference was statistically significant (p<0.001). Some sites were negatively selected in more than one sequence.

### Stop Codons

**Figure 6:**
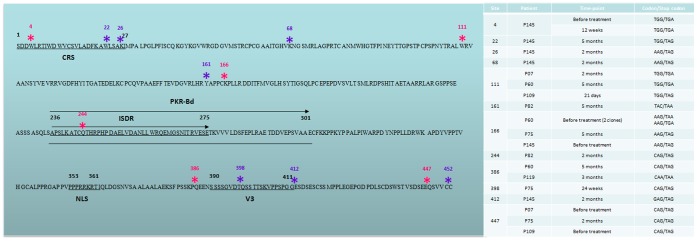
Stop Codon. Graphic representation of sites where stop codons were identified. Pink – more than one occurrence; purple-one occurrence. NZL1 (GenBank accession number D17763) – reference sequence for genotype 3 was used for the graphic representation. CRS – cytoplasmic retention site; PKR-Bd – PKR binding region; ISDR – IFN sensitivity determining region; NLS – nuclear localization signal; V3– variable region 3.

During the analysis of all the sequences generated in this study and considered together with those obtained by Bittar *et al* (2010), 23 occurrences of stop codons were detected [20]. The majority of them, 73.9% (17/23), were found in NR sequences, and no stop codon was identified in SVR. Some of these non-sense mutations were found at the same site in different patients and, in one case (P145), through different time-points. The sites where the stop codons occurred are shown in Figure 6. Most of the stop codons (15) were TAG, followed by TGA (5) and TAA (3). The amino acids that were most associated with mutating to a stop codon were glutamine (Gln) at four sites, and tryptophan (Trp) and lysine (Lys) at three sites each; these mutations correspond to 20 of the 23 mutations found. All of the sites where these codons were found were highly conserved. Some stop codons at the same site in different patient sequences were encoded by different codons (Figure 6).

### Relative Genetic Diversity

**Figure 7:**
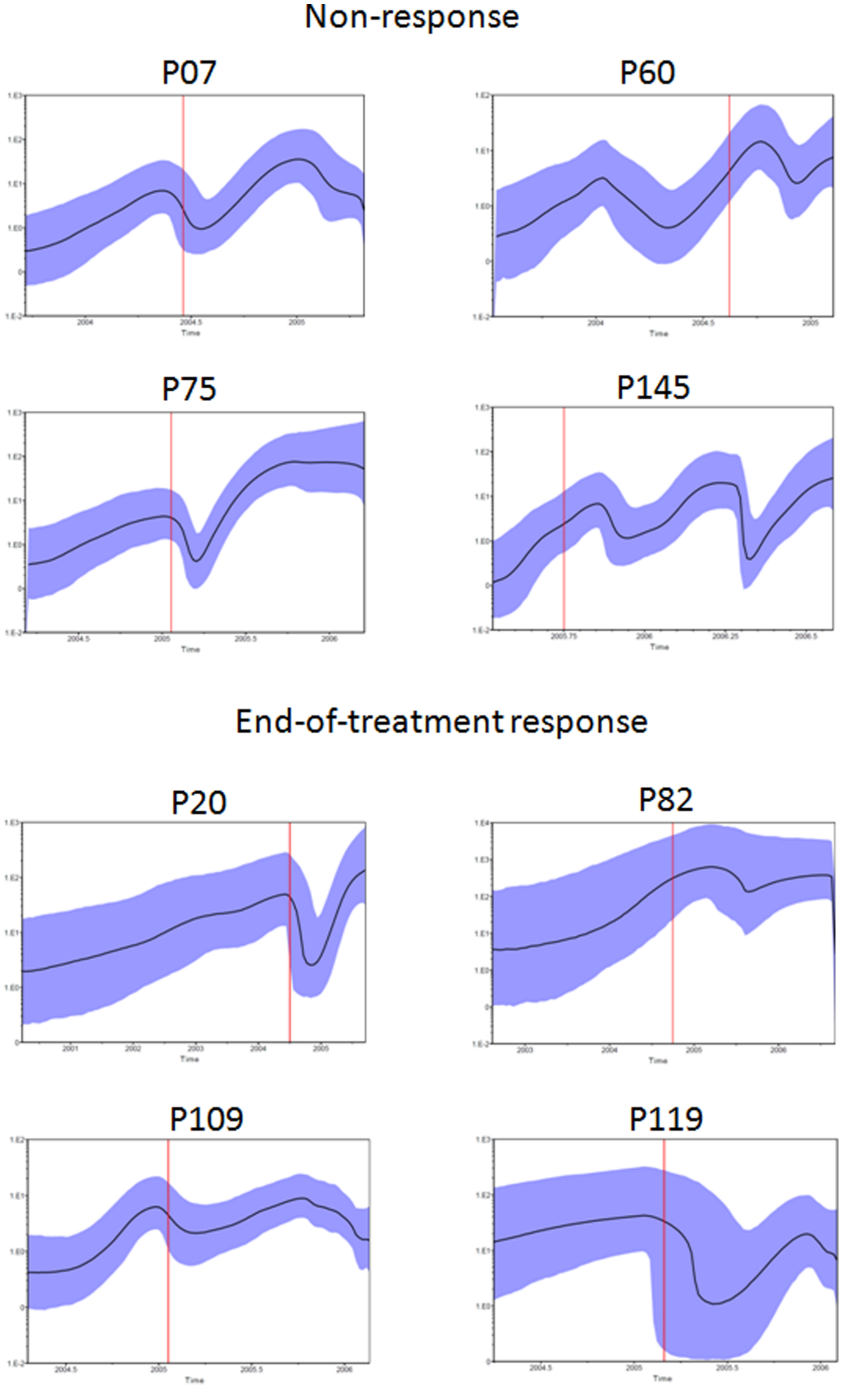
Bayesian Skyride. Skyride plots showing the relative genetic diversity. Black solid lines represent the median posterior distribution, blue shaded areas are the 95% Bayesian credible intervals, vertical red lines represent the approximate time at which the treatment started (BT time-point).

The relative genetic diversity analyses can be seen in the skyride plots shown in Figure 7. In these plots the quasispecies diversity in most patients shows an exponential growth before the beginning of the treatment, except for patient P119, who seems to be constant and patient P60, who shows variation with time. Around the beginning of the treatment (represented by the red line for the approximate BT time-point), all patients show a major oscillation in quasispecies diversity, with the exception of patient P82 samples, which remained almost constant with only a slight variation. Most patients presented a decrease in genetic diversity after the beginning of the treatment (P07, P75, P20, P109 and P119). Patient P145 showed an initial increase in diversity followed by a fluctuation, which is in accordance with the distance data for this patient.

### Phylogenetic Relationships and Correlation with Other Data

**Figure 8:**
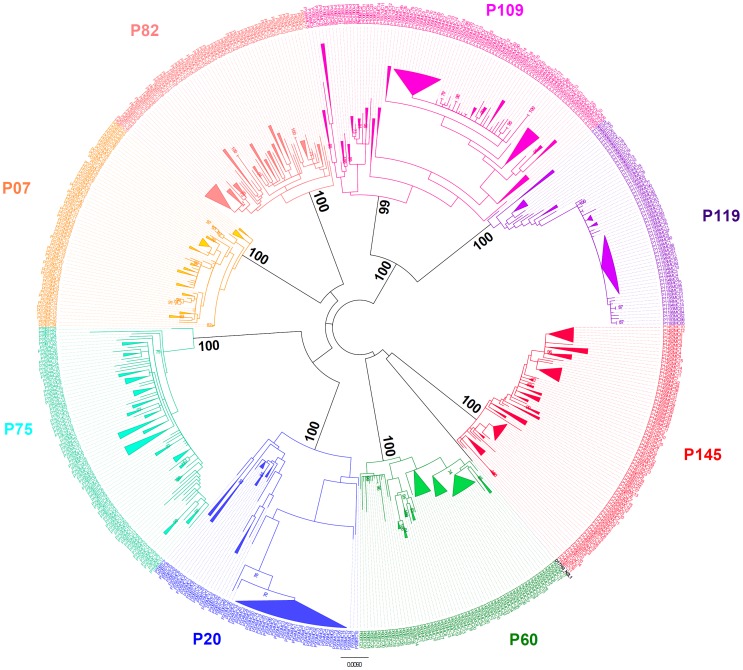
Phylogenetic tree. Unrooted Maximum-Likelihood phylogenetic tree was constructed with HKY85 substitution model including Gamma distribution parameter (HKY+G). Bootstrap was performed with 500 replicates. Values above 70% were considered significant.

A phylogenetic tree was constructed with the sequences from this study together with before treatment sequences from the previous study [20] (Accession numbers: EU826174 to EU826233 and from EU826249 to EU826352), totalizing 600 sequences, and the reference sequence for genotype 3a NZL1 (Accession number: D17763) (Figure 8). All sequences from the same patient grouped on a monophyletic branch with significant supporting values, showing there was no cross-contamination among patients.

**Figure 9:**
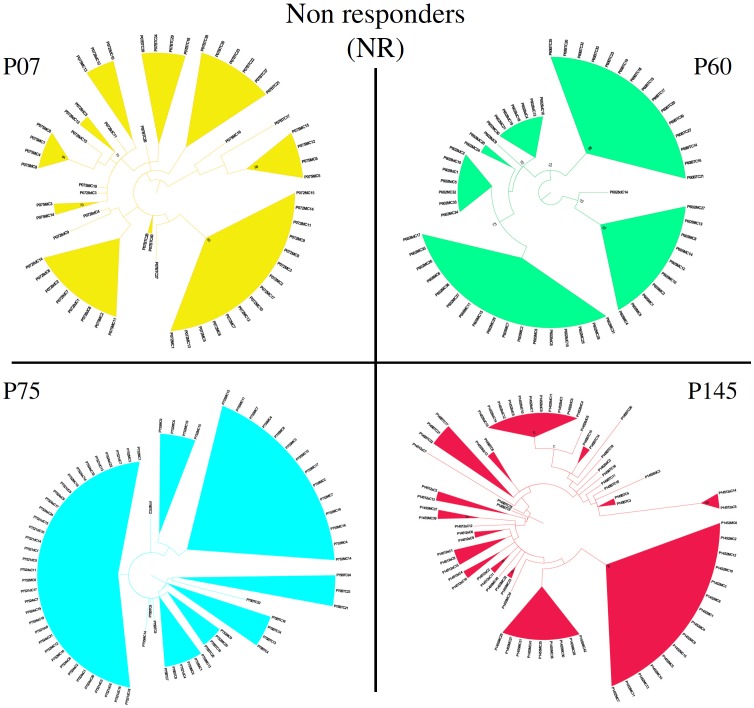
Non-responders individual phylogenetic trees. Unrooted Maximum-Likelihood phylogenetic trees from NR patients were constructed with HKY85 substitution model including Gamma distribution parameter (HKY+G). Bootstraps were performed with 1000 replicates. Values above 70% were considered significant. BT - Before treatment; w - weeks; d - days; m - months.

**Figure 10:**
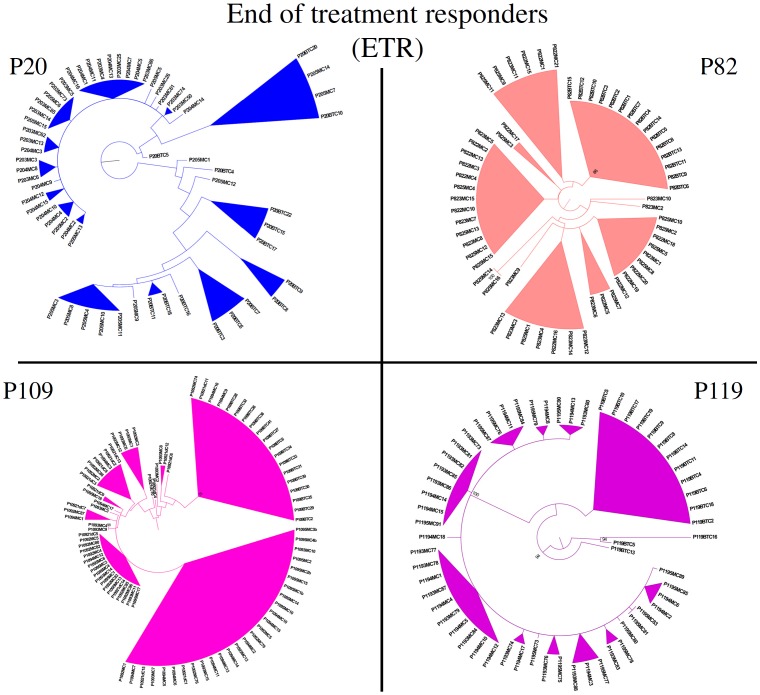
End-of-treatment responders individual phylogenetic trees. Unrooted Maximum-Likelihood phylogenetic trees from ETR patients were constructed with HKY85 substitution model including Gamma distribution parameter (HKY+G). Bootstraps were performed with 1000 replicates. Values above 70% were considered significant. BT - Before treatment; d - days; m - months.

Sequences from P60 and P82 from before the treatment clustered together, away from the other time-points, in a monophyletic branch with bootstrap of 98 and 86 respectively (Figures 9 and 10).

From the phylogenetic tree for patient P20 (Figure10), the viral population at 5 months seems to be derived from two separate before-treatment populations. Interestingly, one of these populations, which was the most prevalent before treatment, is not present in any of the 3 and 4 months time-point samples. This data supports the genetic distance data that show a major decrease in 3 and 4 month time-point sequences when only one population was sampled. At 5 months, the second viral population reappears, which increases the genetic distance. The ω values at 5 months can be explained not by an increase in selective pressure, but is probably a result of an increase in the synonymous changes due to the presence of two different populations. Ultimately patient P20 viral population changed converging towards a previously existing, but not predominant, population and, after failing treatment, both populations were able to reestablish themselves.

The phylogenetic tree also demonstrated some interesting evolutionary behaviors of the viral population of patient P60 (Figure 9). After-treatment sequences from patient P60 were grouped on two different branches with significant support. The first group comprises one sequence from the 28 day time-point, one at 2 months, and nine sequences from the 5 month time-point. The second group, which contains all other P60 after-treatment sequences time-points together with the six remaining sequences from the 5 month time point, grouped on a terminal monophyletic branch along with three sequences from the 28 day time-point and eight sequences from 2 months. The first group had a low prevalence in the first two time-points after treatment, having only one strain in each, but became the most prevalent at the last time point. This scenario corroborates the genetic distance results where P60 showed an increase at 5 months, probably due to the existence of quasispecies that evolved from two different lineages.

The phylogenetic tree for patient P07 shows all of the sequences from the 2 month time-point clustering as a monophyletic group, with a bootstrap of 93, indicating a recent common ancestor with a before-treatment strain (Figure 9). All samples from the 28 day time-points are clustered in two different branches together with some 5 month strains. The remaining strains from the 5 month time-point are grouped on a third outer monophyletic branch separated from all other after-treatment time-points that share a common ancestor with a before-treatment strain. These data explain why the genetic distance rises in the fifth month (Figure 2).

Phylogenetic data on patient P109 show that sequences clustered on two major branches (Figure 10). This indicates that the majority of the after-treatment population is derived from a population that is not sampled in a before- treatment time-point. Conversely, all after-treatment strains from patient P119 are derived from the before-treatment samples.

## Discussion

This study characterizes hepatitis C evolution, based on NS5A protein, throughout treatment in patients infected with HCV genotype 3a who show different treatment outcomes.

The high mutation rate of RNA viruses, such as HCV, provides a pool of closely related variants, the quasispecies, which offers the virus with many possibilities for evading the immune system and therapy. It is interesting that in two of the non-response patients, the same variants that were sampled before the treatment were also sampled during (P75) or after treatment (P145). This is unlikely to occur randomly since these samples were collected more than six months apart, and HCV is estimated to produce 10^12^ copies of its genome per day [21] with a high mutation rate, suggesting that these strains provided some advantage to the virus enabling it to survive the treatment, and were therefore selected.

In contrast, the virus circulating in end-of-treatment responder patients was not initially successful in evading the treatment, so the virus was not detected using the PCR technique. Presumably, in order to reestablish the infection, it replicates at low rates and at some point produces strains with a better fitness to the new condition, and because they are capable of evading the treatment, are selected, resulting in the relapse. After the best adapted pool of strains for the new condition has been selected, the different sequences would be expected to converge to the most favorable genetic composition for survival. This is the case for patients P109 and P119. In the distance data at the time-point where a predominant strain arises, the genetic divergence between the sequences falls, even though it still has a low frequency. It is also expected that through time the virus population would recover its variability.

The analysis of selective pressure by site showed some sites that are negatively selected in more than one patient, suggesting a functional constraint of the protein. However, as this does not apply for all patients, and one site, site 408, was both positively and negatively selected in two different patients (P109 and P20, respectively), it seems that evolutionary processes are different in each patient and are guided by the interaction of virus and host. Viral samples from patients who had an end-of-treatment response have more sites undergoing negative selection than samples found in non-responder patients, and this difference is statistically significant. Since this group passed through a recent bottleneck due to a strong selective event (Interferon/Ribavirin administration), it probably conserved the amino acids that proved most effective in evading both treatment and the immune system, thus allowing the infection to be reestablished. It is interesting, however, that the ETR group, despite having more negatively selected sites, presented an overall ω close to that of the NR group. A possible explanation for this is that the remaining sites have more relaxed constraints.

The occurrence of stop codons at the same site in different patients, and in one case persisting through all time-points of the same patient, is quite interesting. One explanation could be that during translation the ribosome jumps or reads through the stop codon. However, this hypothesis does not explain why they are being found in the same site in different patients. Since NS5A is a multifunctional protein and its functions are not fully understood, the proteins resulting from this RNA that encode stop codons could have a specific function during the infection, different from the one achieved by full-length NS5A. One of the known functions of NS5A is an important role in viral replication, and it has been shown that defective NS5A impairs HCV replication. Though it is also known that NS5A is the only HCV protein that can act in *trans* complementation, meaning it is acting on a viral RNA other than the one from which it has been translated [22], [23]. These characteristics make it possible that the defective genomes could persist by being encapsulated with the help of normal NS5A acting in *trans.* Other studies have shown that defective genomes circulate during HCV infection and also during other *Flavivirus* infections [24], [25], [26]. Another point to consider is that having NS5A sequences with stop codons do not seem to be disadvantageous for viral survival. No nonsense mutations were identified in sequences from SVR patients, while 73.9% of them were found in sequences from non-responders [20]. These defective variants could give the virus some advantage and play an important role in evading the therapy response. Another issue that suggests some kind of viral fitness enhancement by these mutations is that most of the stop codons that were found at the same site in different patients were not encoded by the same codon. For example, site 111 (originally TGG) mutated into three different stop codons in three different patients, two of them changed to TGA and the other to TAG. In the case of site 166 (originally AAG), two clones from the same patient and same time-point presented two different stop codons (TAA and TGA), and in two other patients the nonsense mutation was due to TAG. This site (166) presented all three codons that stop translation. Site 386 had mutated from CAG to TAG in patient P60 at 5 months and from CAA to TAA in patient P119. These sites converged to nonsense mutations in different ways both in the same patient and in different patients, suggesting that stopping translation at these points may provide some kind of evolutionary advantage to the virus.

### 

#### Some insights on evolution

Well-adapted viruses do not kill their hosts, at least not in the short term, as by doing so they would ultimately be killing themselves and have less time to spread viral particles to other hosts. The Human Immunodeficiency Virus (HIV), for example, has adapted to infect, survive and replicate in humans, but it also can kill its host very quickly. As HIV is considered a virus of recent origin, thought to have been introduced into humans between 1884 and 1924 [27], it has probably had insufficient time to establish the most adapted scenario through co-evolution. The hepatitis C virus, on the other hand, can be considered a successful virus in evolutionary terms. It causes a persistent infection and remains silent, presenting no clinical symptoms, for decades. Hepatitis C virus genotypes have been co-evolving with the human host for a long time; they are thought to have diverged 500 to 2000 years ago, leading to a better fitness [28].

The samples used in this study were obtained from chronically infected patients who had been through interferon and Ribavirin treatment and had not responded. These viruses not only evaded the host immune system, but also evaded therapy. What can be seen in this study is that, although HCV is adapted to infect *Homo sapiens* in general, each individual specimen represents a different environment, leading to different evolutionary processes. In some cases persistence is achieved by strains that were not initially the best adapted for survival, and therefore were not sampled before the treatment started, but after a new selective environment – the treatment - was introduced the strain gained fitness. The opposite can also be found, where all after-treatment strains are derived from a unique before-treatment population or viral population changed by converging to a previously existing, but not predominant, population and, after evading treatment, both populations were reestablished. Finally, the after-treatment strains were all derived from before-treatment samples in some patients.

Despite the differences in population dynamics of each host, this analysis show that before-treatment samples from most of the non-responder patients (except P60) presented a viral population that persisted after treatment, suggesting they were adapted to evade the treatment. In contrast, the population found in before treatment samples from most ETR patients (except P119) either are selected out or appears in low frequency after relapse, therefore changing the population structure. The exceptions illustrate the uniqueness of the evolutionary process, and therefore the treatment resistance process in each patient. No specific relationship between genetic composition and therapy response could be established.

### Conclusion

Hepatitis C virus is a highly variable virus, which confers a range of possibilities for it to evolve and adapt to new conditions. The evolution of this virus during and after treatment is linked to the environment to which it is subjected, that is the patient. The analysis of evolutionary behavior throughout treatment showed that each patient presented different population dynamics unrelated to therapy outcome. However it seems that the viral population from non-responders that resists the treatment already had strains that could evade therapy before it started. No specific pattern was found in viral strains that could determine therapy response. Although this is a small sample size study it shows there appears to be different mechanisms that should be further studied in larger studies.

## Methods

### Ethics Statement

This study was approved by the Ethics Committee of the Hospital de Base from São José do Rio Preto, and all participants signed an informed consent.

### Population and Samples

In order to address the evolutionary pattern of the NS5A region in HCV genotype 3a patients throughout treatment, blood samples were collected from genotype 3a-positive patients at the Blood Center of São José do Rio Preto, State of São Paulo, Brazil. The collection of the samples was scheduled to be performed before treatment, during (12 weeks or 24 weeks) and after treatment (21 days, 28 days, 2 months, 3 months, 4 months and 5 months) to provide treatment response data and a complete overview of the evolutionary behavior. Patients were classified into three groups according to treatment response: sustained virological responders (SVR), i.e. the virus was not detected after treatment or during the 5-months follow-up; non-responders (NR), since they showed no virological response; and end-of-treatment responders (ETR), i.e. a virological response was detected, but during the 5-months follow-up there was a relapse. Owing to availability of patients, some samples could not be collected since the patient did not attend the facility on the day it was schedule. In order to establish the population of study patients with a history of alcoholism and co-infection with other agents that could cause liver damage were excluded. Also, patients from the three response groups that had a more complete set of collections according to the pre-determined time-points were chosen. The final study population is 4 SVR patients, 4 NR patients and 4 ETR patients. Samples from the SVR group and before treatment samples from most patients enrolled in this study were analyzed in a previous study and were used in this study for comparative analyses of the evolutionary behavior (**EU826189 to EU826218; EU826249 to EU826263; EU826294 to EU826307**) [20]. The exception was patient P82; before-treatment samples from patient P82 were analyzed in this work as they had not been analyzed in the previous study. Treatment consisted of Interferon-α (IFN-α) and Ribavirin administration for 24 weeks. Based on the samples from during and after treatment time-points that were available the ones that would be used in the analysis were determined. Time-points for each patient were considered as presented in Figure S1.

### Extraction of HCV RNA and Amplification of the NS5A Region

Viral RNA was extracted from blood serum samples using a QIAamp Viral RNA Mini Kit (QIAgen), and cDNA was synthesized using a High-Capacity cDNA Archive Kit (Applied Biosystems) according to the manufacturer’s instructions. For the PCR and NESTED-PCR reactions, three sets of forward and reverse primers for the NS5A region were designed (Table S2, supplementary material). The amplification mix for both PCR and NESTED-PCR reactions contained 1 µl (5 U) of a proofreading polymerase (Long PCR Enzyme Mix; Fermentas) together with 5 µl of 10X Long PCR Buffer with MgCl_2_, 4 µl of DMSO, 1 µl of dNTP (10 mM), 1 µl of each primer (20 pmol), 10 µl of synthesized cDNA for the PCR reaction and 5 µl of PCR product for the NESTED-PCR reaction, plus nuclease-free water provided with the enzyme kit to a final volume of 50 µl. The amplified products were analyzed on a 1% agarose gel.

### Cloning and Sequencing

Cloning was performed using a TOPO XL Cloning TM Kit (Invitrogen). Fragments of 15 clones at each time-point from each patient were purified using a GeneJET Plasmid Purification Kit (Fermentas). The entire NS5A region was sequenced using eight primers: the vector primers *M13F* and *M13R*, and six inner primers: three sense and three anti-sense (Sense: *H.NS5AI-F1, H.NS5AI-F2* and *H.NS5AI-F3*, antisense: *H.NS5AI-R1*, *H.NS5AI-R2* and *H.NS5AI-R3*) [20]. The sequencing reaction was performed with BigDye Terminator (Applied Biosystems) and the products were sequenced in an ABI 3130XL sequencer (Applied Biosystems). The reaction mixture consisted of 2.2 µl of Milli-Q autoclaved water, 2 µl of 5× Sequencing Buffer (Applied Biosystems), 0.8 µl of primer (5 pmol/µl), 2 µl of Big ET Dye Terminator, plus 3 µl of sample. Sequencing reactions consisted of a “hot start “step of 10 min at 95°C, followed by 96°C for 1 minute, then 25 cycles of 96°C for 15 s, 50°C for 15 s and 60°C for 4 min.

### Sequence Analysis

In order to analyze viral evolution over time, sequences from samples collected before treatment, and previously published (Accession numbers: EU826174 to EU826233 and from EU826249 to EU826352), were also used in the analyses [20]. The sequences obtained in this study were subjected to BioMol - Electropherogram quality analysis (http://adenina.biomol.unb.br/phph/) [29], a phred phrap [30], [31] analysis site, for quality checking and contig construction. The contigs obtained for each clone were aligned, along with the reference sequence NZL1 for genotype 3 (GenBank accession number D17763), using *Clustal W* software nested in the BioEdit 7.0.9.0 package [32], [33]. All sequences were edited on *Bio Edit* [32] to remove the vector fragments, leaving only the complete sequence of the NS5A region.

### Evolutionary Analysis

Quasispecies were analyzed using *LOCSPEQ 1.0* software [34]. The genetic distances were calculated using MEGA 5.0 software by the number of base differences per site from between sequences within each time point [35]. Results were plotted in a graphic for each patient (Figure 2). The overall dN/dS ratios (ω) were calculated with HyPhy 2.0 using MG94xHKY85_3x4 substitution model and global parameters. Site per site ω was calculated by Single Likelihood Ancestor Counting (SLAC) method using HyPhy [36]. Bayesian skyride plots were performed using the BEAST package [37]. Phylogenetic analysis was performed using *PhyML* [38]. A Maximum-Likelihood tree was constructed using the HKY85 substitution model including a Gamma distribution parameter (HKY+G). Bootstrap was performed with 500 replicates for the tree containing all the sequences and 1000 replicates for individual trees from each patient. Values above 70% were considered significant.

### Statistics

Statistical analyses for the genetic distance and ω data were performed by Mann-Whitney tests, and a Chi-square test was used for negatively selected sites. p-values lower than 0.005 were considered significant.

## Supporting Information

Figure S1 Graphic representation of the time-points used for treatment follow-up. The red circles represent the time-points when the sequences were used for the analysis from this study. Before treatment sequences from P07, P20, P60, P75, P109, P119, P145 were from a previous study detailed in Bittar et al (2010).
(TIF)

Table S1 Mann-Whitney statistical test on distances between each time-point. A. P07; B. P60; C P75; D. P145; E. P20; F. P82; G. P109; H. P119. In red differences that were not significant (significance p<0.005).
(TIF)

Table S2 Primers used on PCR and NESTED-PCR reactions. *published in Bittar et al (2010).
(TIF)
