# Supplementary figures and images for: On Hepatitis C Virus Evolution: The Interaction between Virus and Host towards Treatment Outcome
Source: PLoS One. 2013 Apr 25;8(4):e62393. doi: 10.1371/journal.pone.0062393 (PMC3636177; doi:10.1371/journal.pone.0062393)

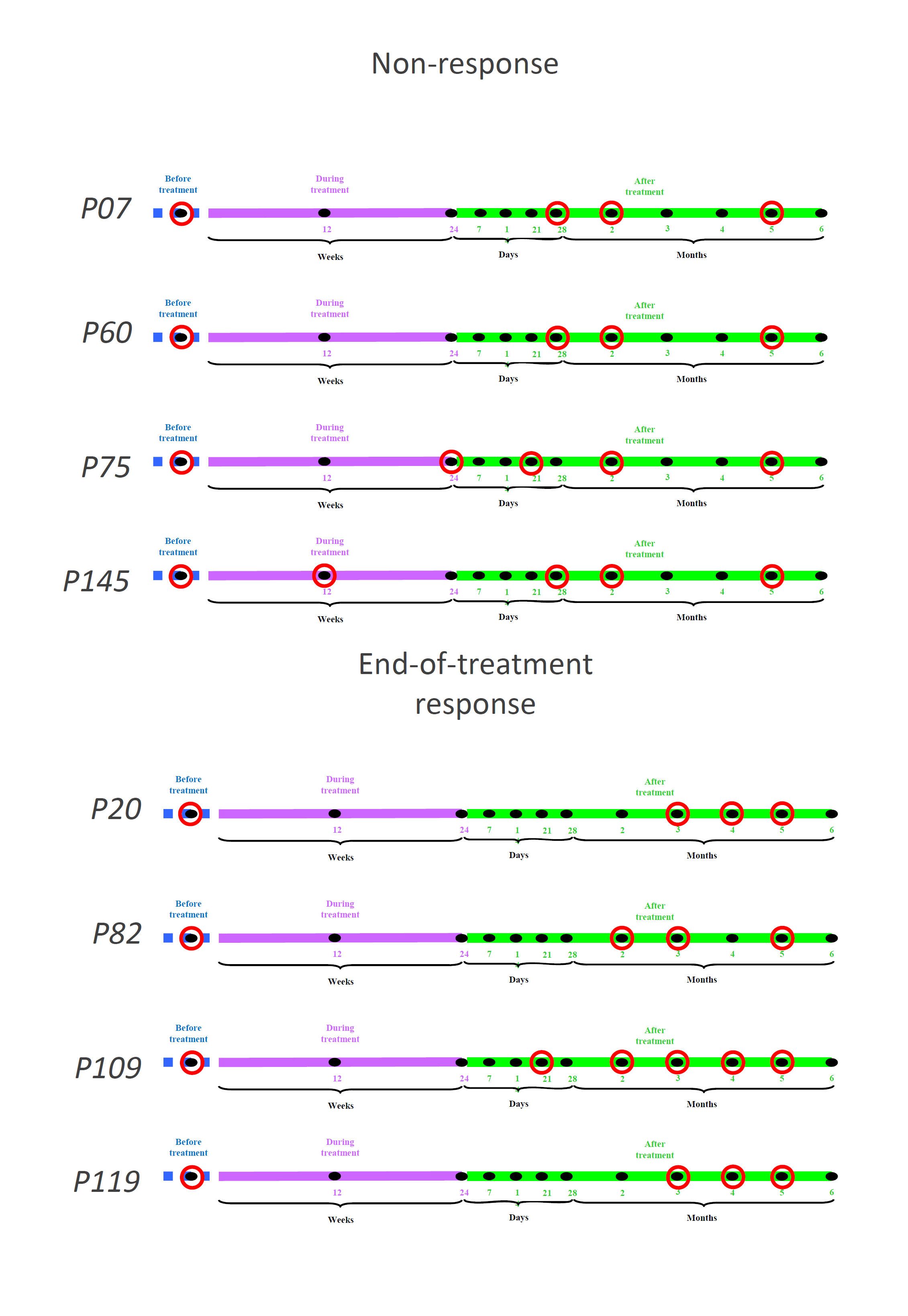

Supplement: Figure S1 — Graphic representation of the time-points used for treatment follow-up. The red circles represent the time-points when the sequences were used for the analysis from this study. Before treatment sequences from P07, P20, P60, P75, P109, P119, P145 were from a previous study detailed in Bittar et al (2010). (TIF) [file pone.0062393.s001.tif]

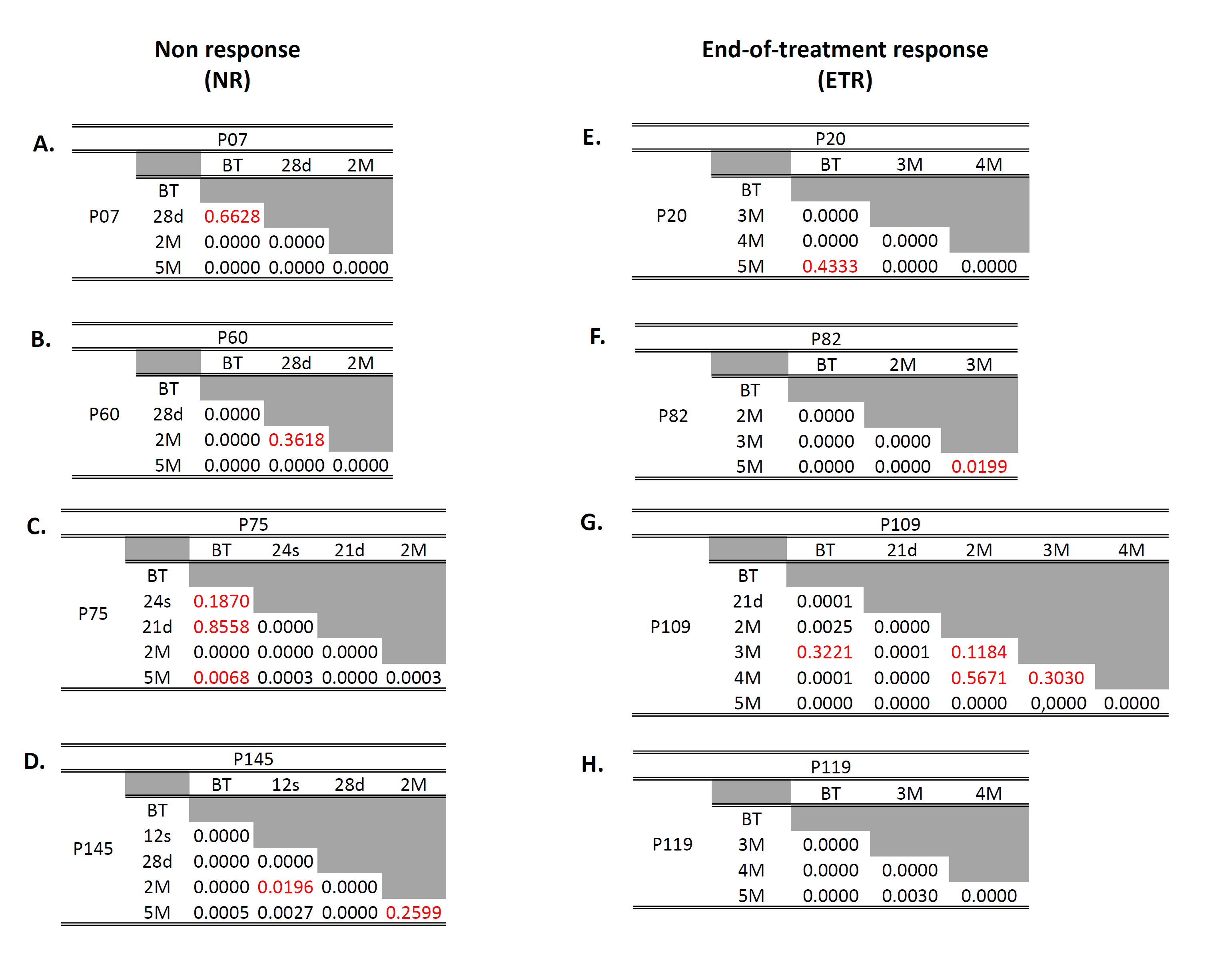

Supplement: Table S1 — Mann-Whitney statistical test on distances between each time-point. A. P07; B. P60; C P75; D. P145; E. P20; F. P82; G. P109; H. P119. In red differences that were not significant (significance p<0.005). (TIF) [file pone.0062393.s002.tif]

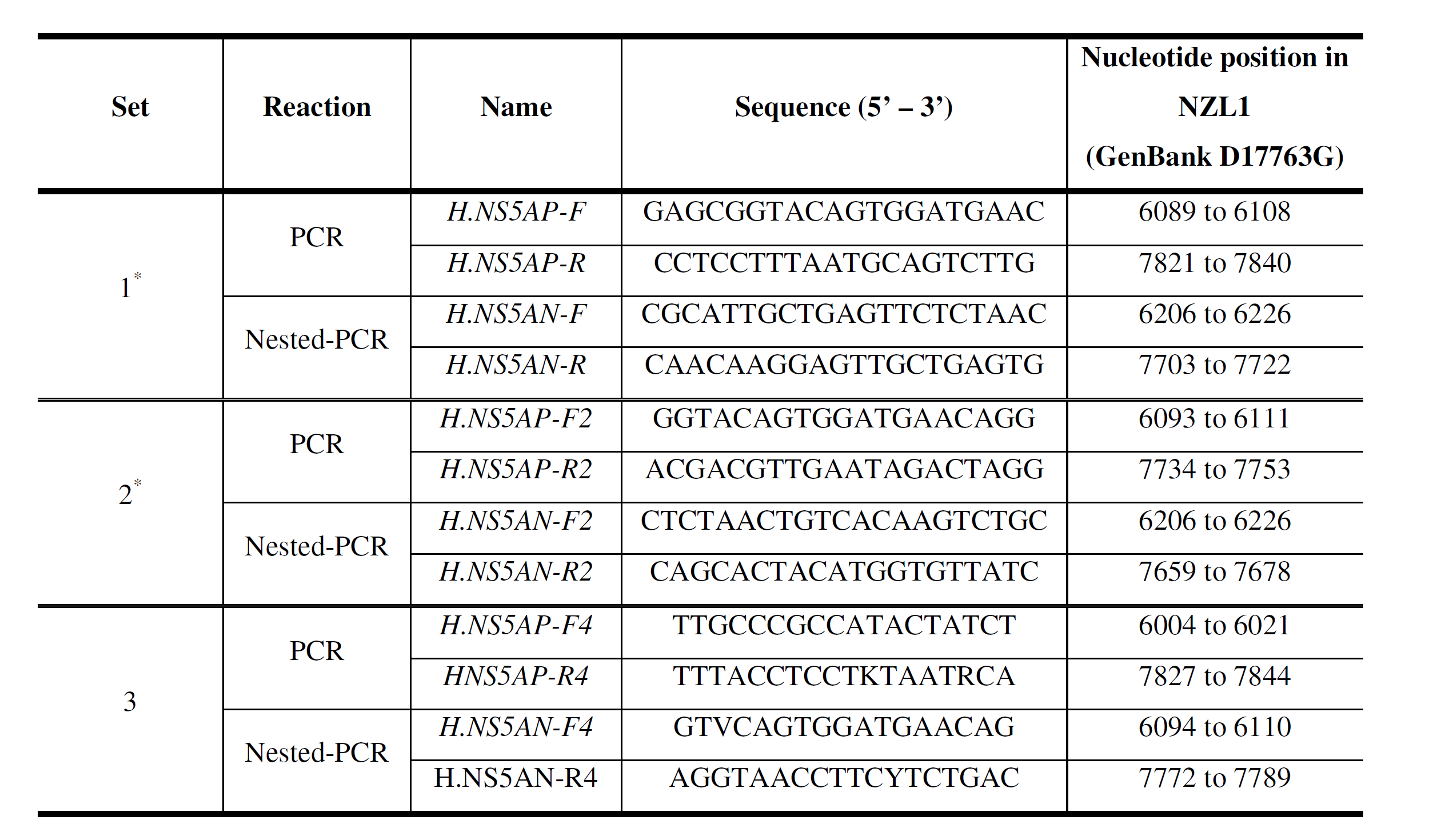

Supplement: Table S2 — Primers used on PCR and NESTED-PCR reactions. *published in Bittar et al (2010). (TIF) [file pone.0062393.s003.tif]
